# Supplementary material for: Chemically programmed STING-activating nano-liposomal vesicles improve anticancer immunity
Source: Nat Commun. 2023 Jul 31;14:4584. doi: 10.1038/s41467-023-40312-y (PMC10390568; doi:10.1038/s41467-023-40312-y)
Supplement: Supplementary file 1 — Supplementary Information [file 41467_2023_40312_MOESM1_ESM.pdf]

Supplementary Materials for

Chemically programmed STING-activating nano-liposomal vesicles improve anticancer immunity

Xiaona Chen,[1] Fanchao Meng,[1] Yiting Xu,[1] Tongyu Li,[1] Xiaolong Chen,[1] and Hangxiang Wang,*,[1,2]

[1]The First Affiliated Hospital, NHC Key Laboratory of Combined Multi-Organ Transplantation, Collaborative Innovation Center for Diagnosis and Treatment of Infectious Diseases, State Key Laboratory for Diagnosis and Treatment of Infectious Diseases, Zhejiang University School of Medicine, Hangzhou, Zhejiang Province, 310003, P. R. China.

[2]Jinan Microecological Biomedicine Shandong Laboratory, Jinan, Shandong Province, 250117, P. R. China.

*Corresponding author. Email: wanghx@zju.edu.cn

This PDF file includes:

Figs. S1 to S30

Table S1

Supplementary Methods

Supplementary figures and tables

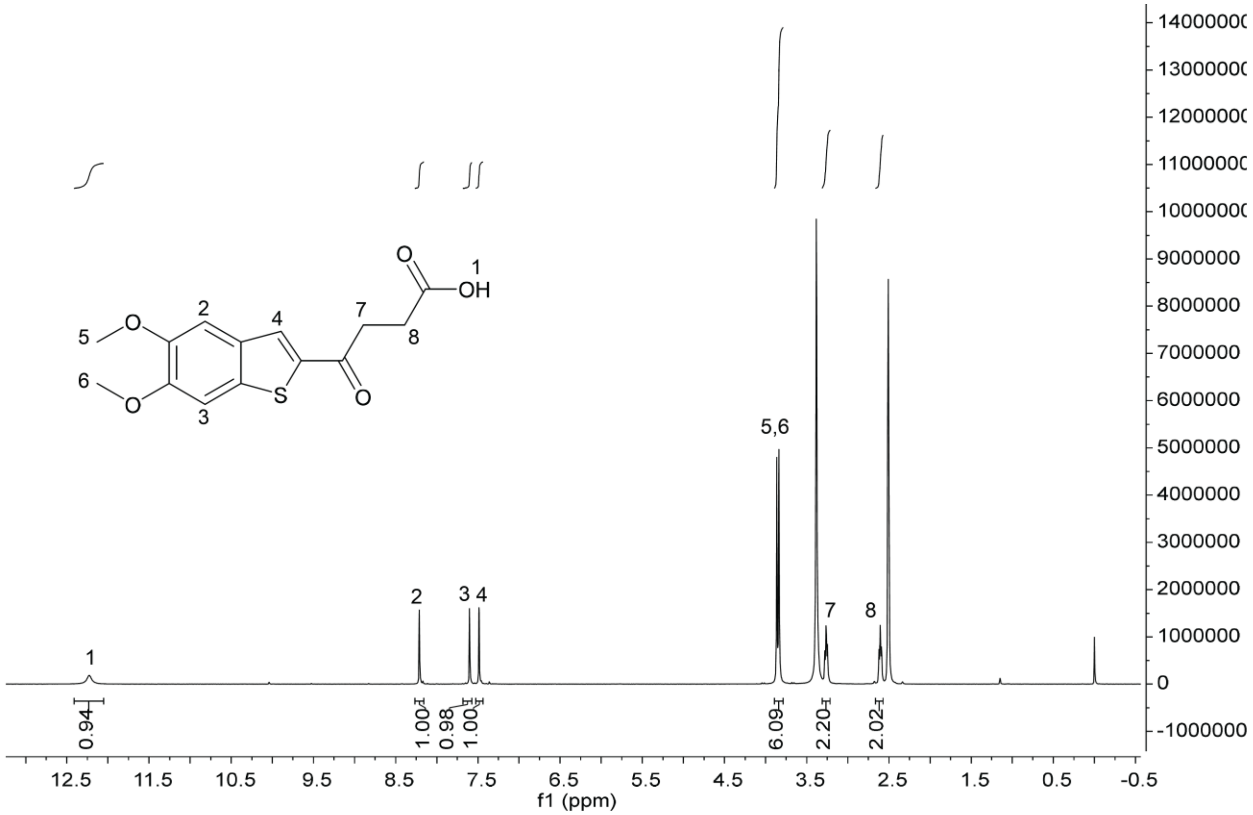

Fig. S1. ^{1}H NMR spectrum of MSA-2 in DMSO-*d*6.

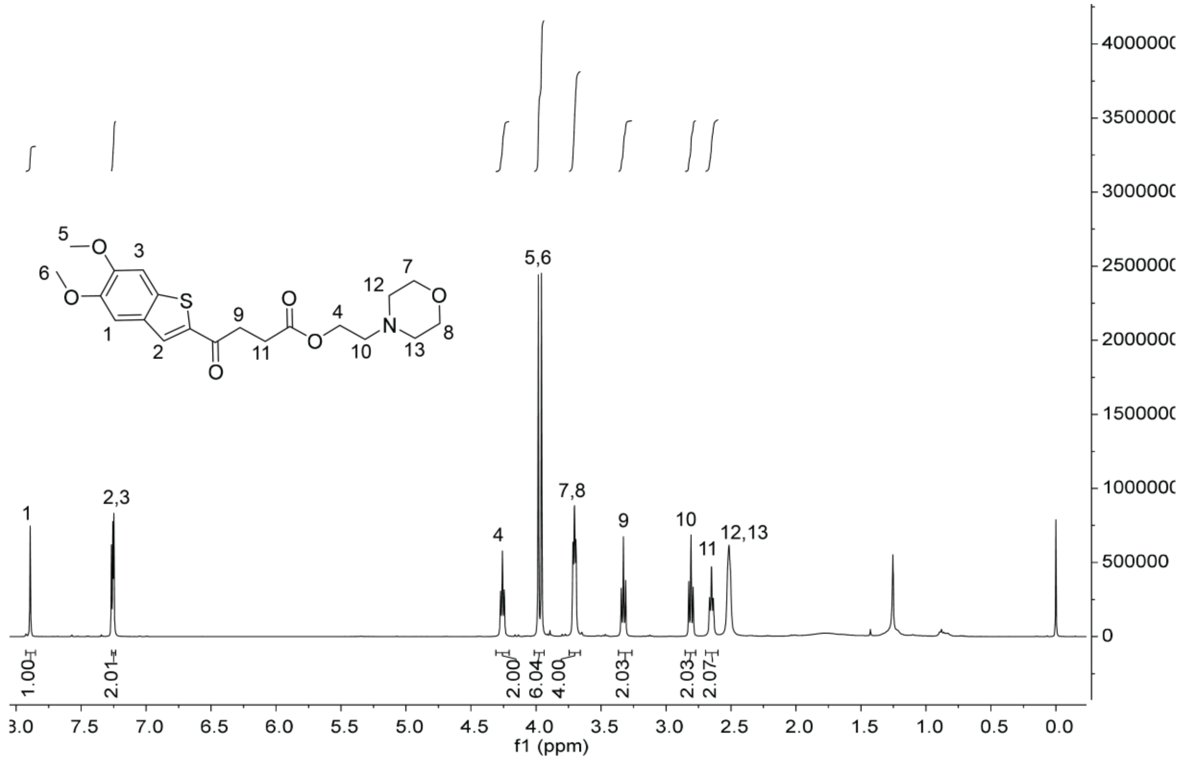

Fig. S2. ^{1}H NMR spectrum of pro-drug **1** in $CDCl_3$.

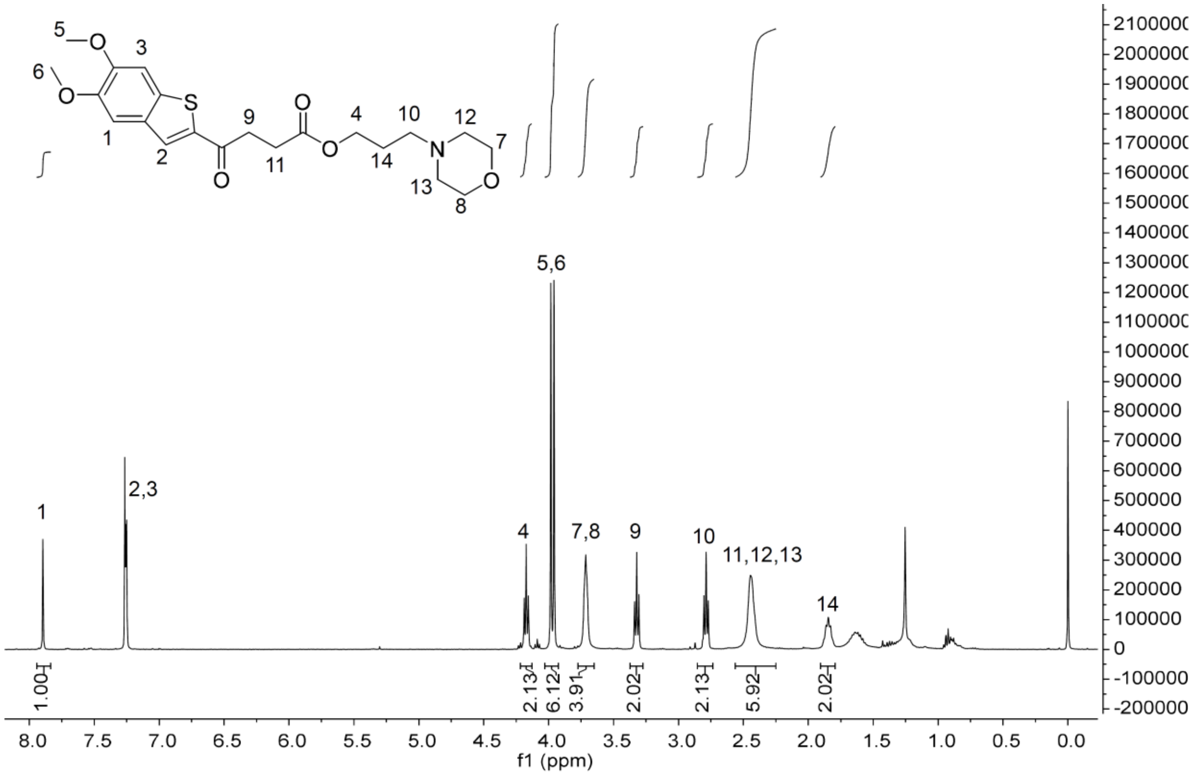

Fig. S3. ^{1}H NMR spectrum of pro-drug **2** in $CDCl_3$.

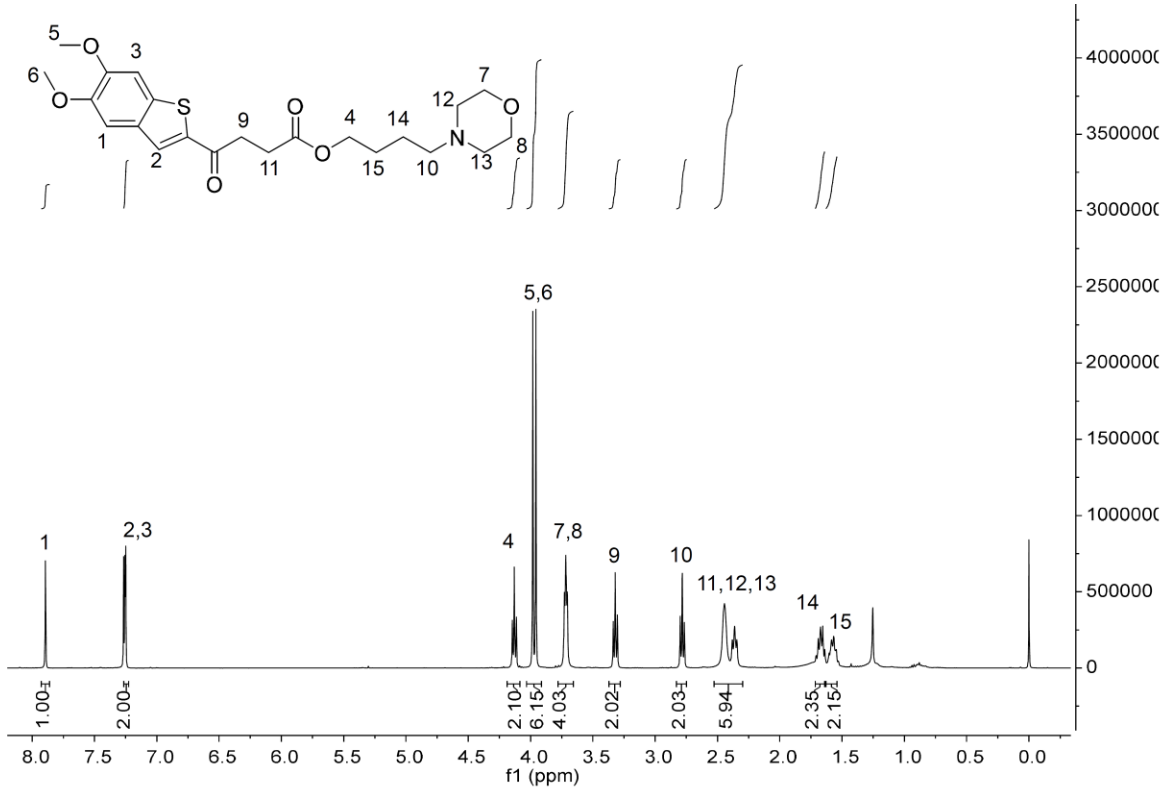

Fig. S4. ^{1}H NMR spectrum of pro-drug **3** in $CDCl_3$.

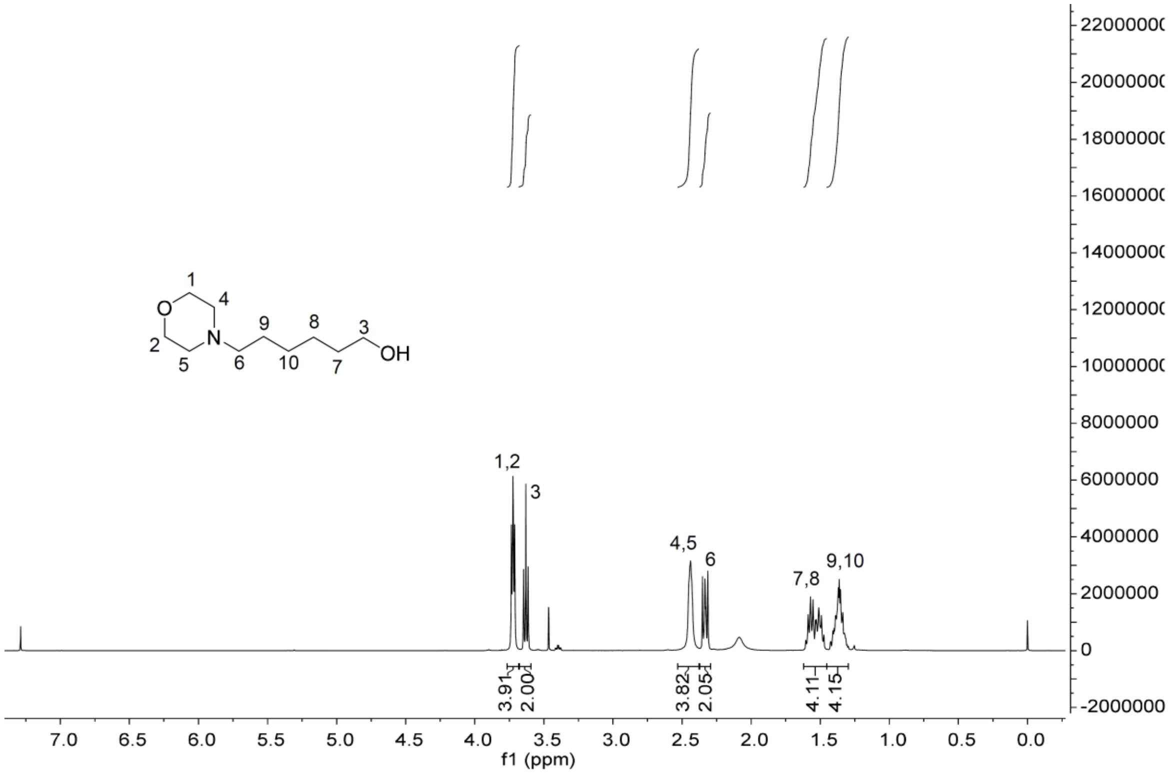

Fig. S5. ^{1}H NMR spectrum of 6-morpholino-1-hexanol in $CDCl_3$.

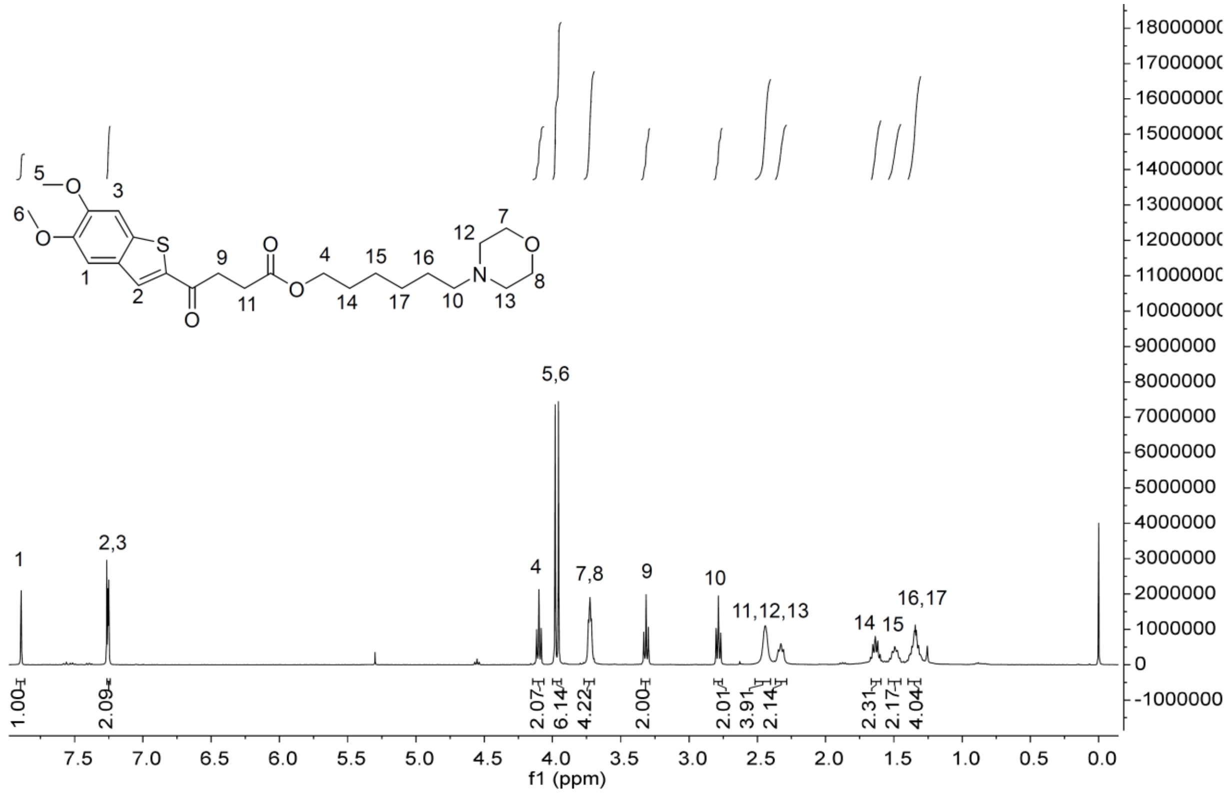

Fig. S6. ^{1}H NMR spectrum of pro-drug **4** in $CDCl_3$.

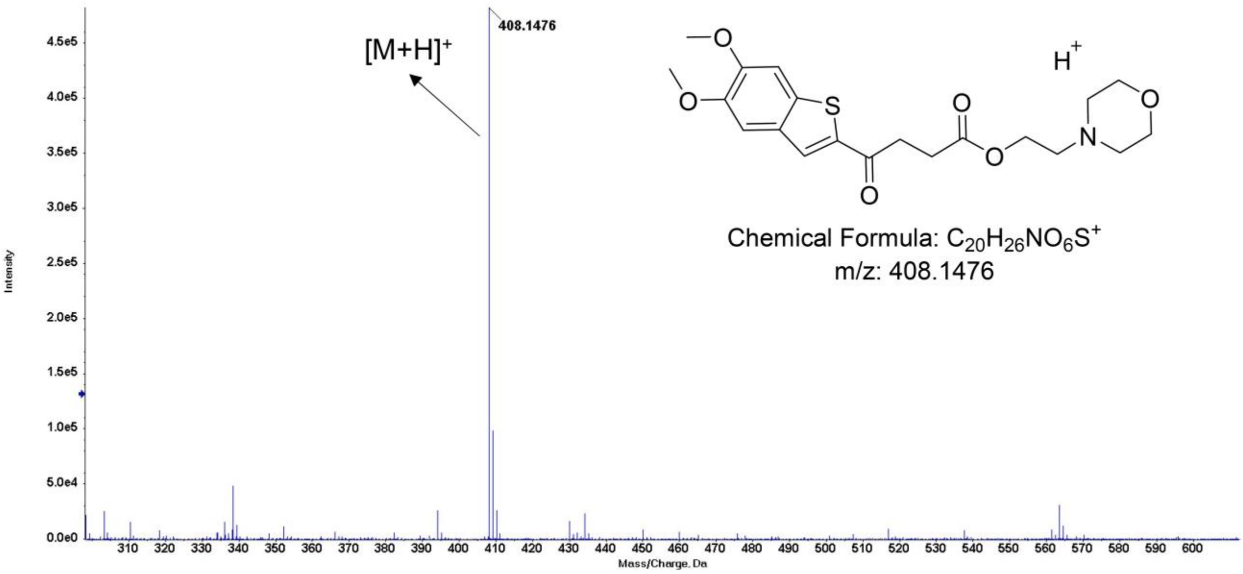

Fig. S7. ESI-MS spectrum of pro-drug **1**.

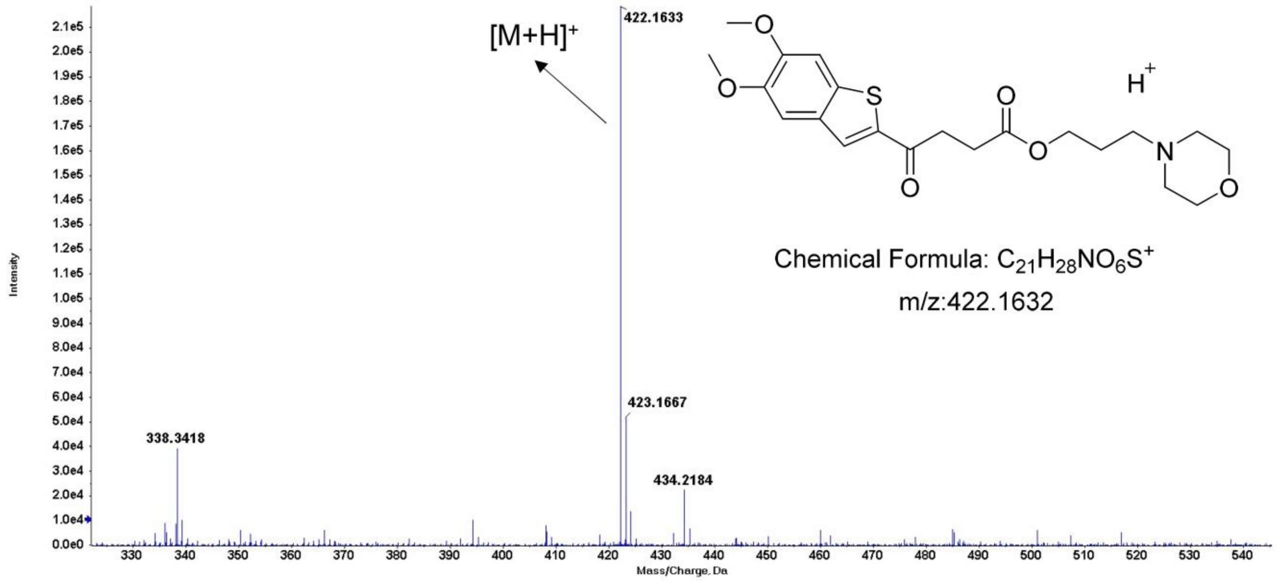

Fig. S8. ESI-MS spectrum of pro-drug **2**.

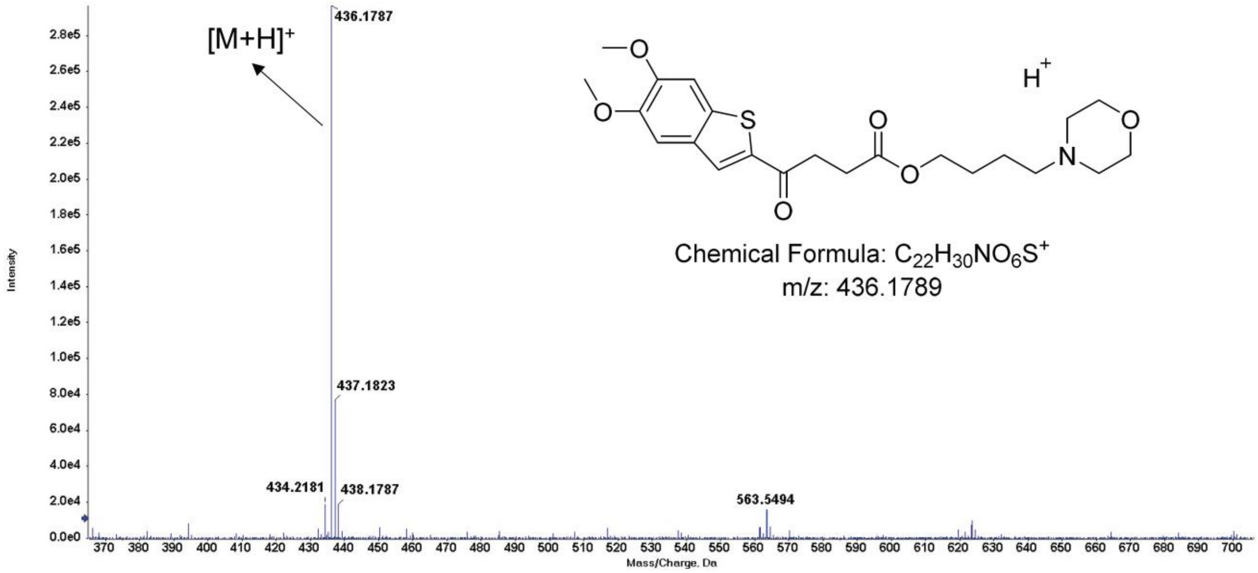

Fig. S9. ESI-MS spectrum of pro-drug **3**.

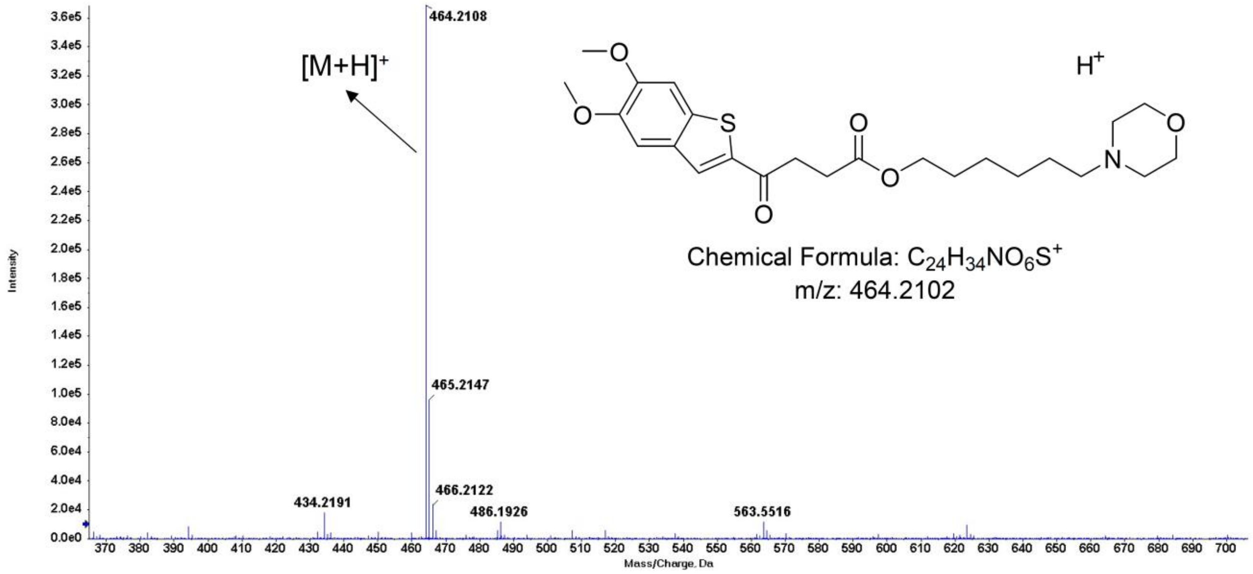

Fig. S10. ESI-MS spectrum of pro-drug **4**.

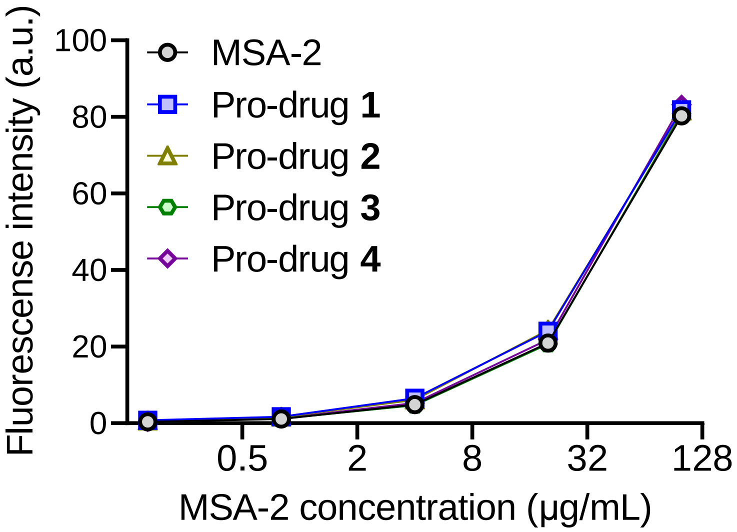

Fig. S11. Plot of fluorescence intensities for different compounds including free MSA-2, pro-drug **1-4** dissolved in DMSO at different concentrations. Ex = 405 nm, Em = 450 nm.

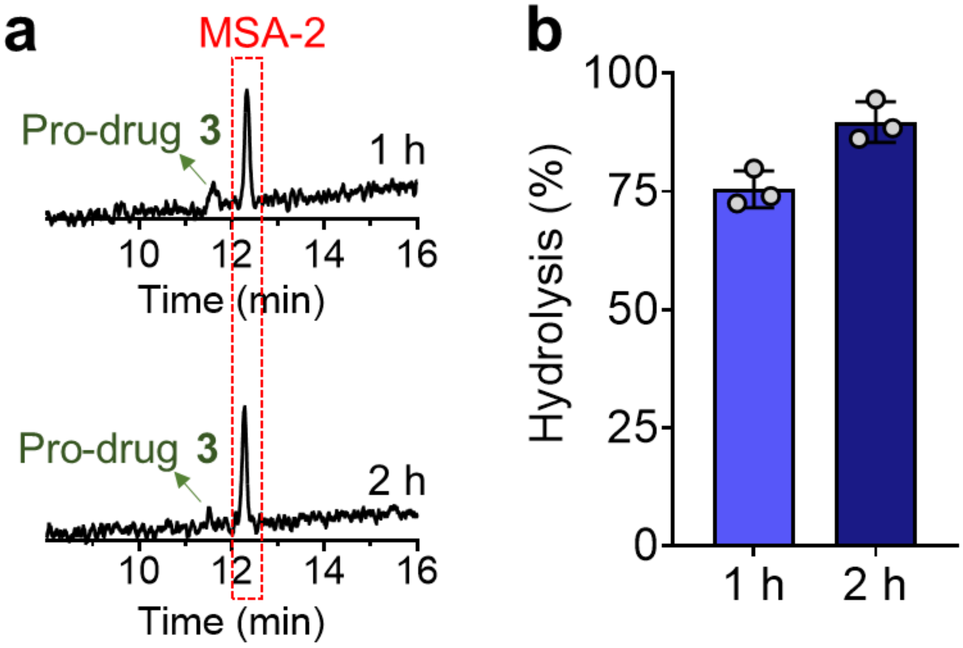

Fig. S12. a HPLC analysis was used to measure drug activation in THP1 cells. The cells were incubated with SAProsome-3 for either 1 or 2 hours. **b** Percentages of activated MSA-2 in THP1 cells were determined using their corresponding standard curves. The data are presented as the mean ± SD (n = 3).

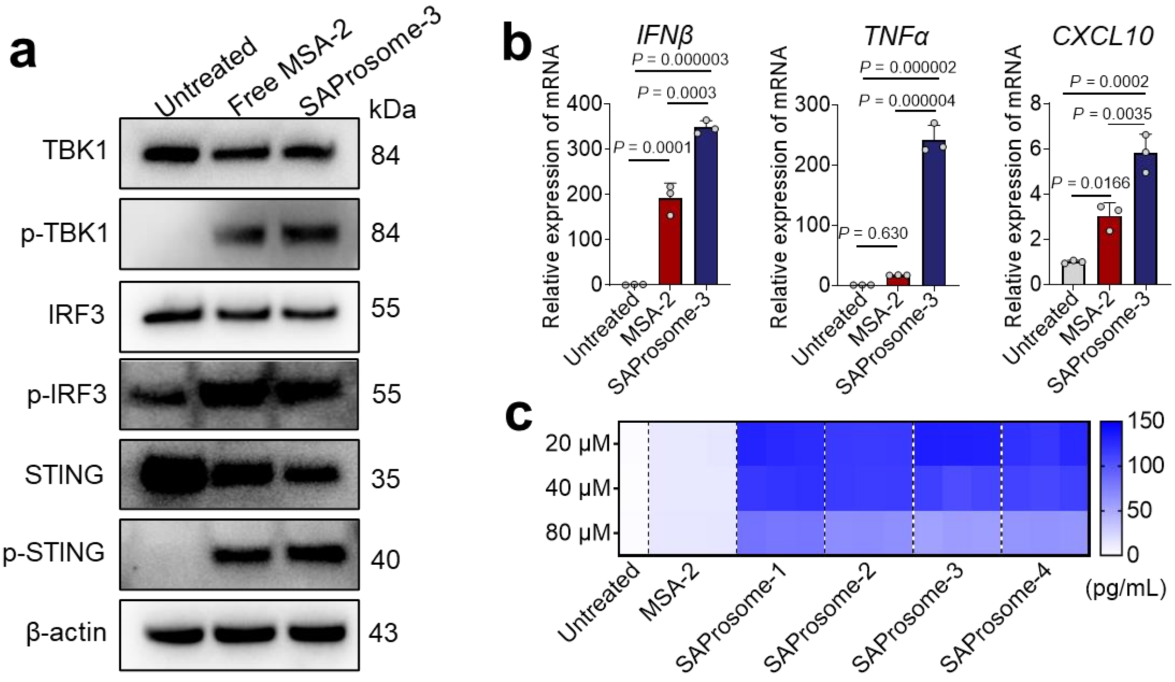

Fig. S13. a–**b** Human THP1 cells were incubated with either free MSA-2 or SAProsome-3 for 6 hours. The activation of the STING pathway was further analyzed using western blot analysis **a** and quantitative real-time PCR analysis **b** of gene expression (n = 3 biologically independent samples). Data are presented as the mean ± s.d. and statistically analyzed using one-way analysis of variance. **c** IFN-β levels in cell culture media of human THP1 treated with the indicated concentrations of MSA-2 formulations were determined by ELISA.

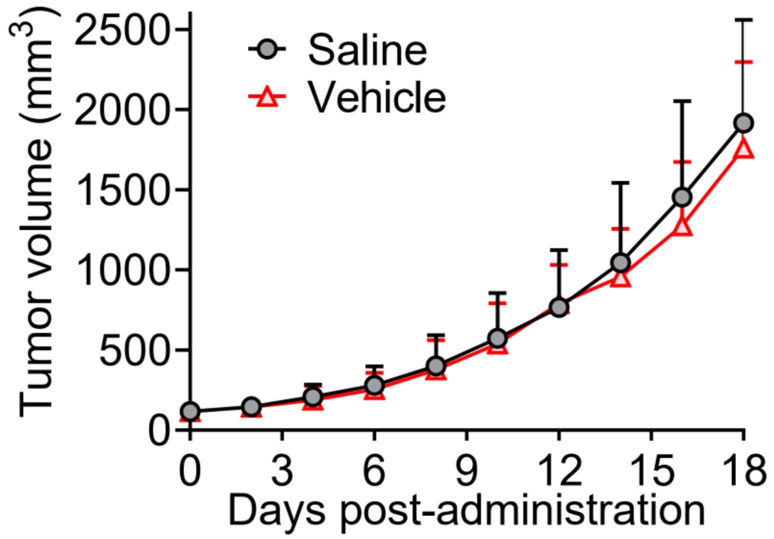

Fig. S14. Tumor growth curves of MC38 xenografts in mice (n = 4/group). Mice were treated with either saline or empty liposomes without pro-drug payloads. Data are presented as ± s.d. of mean.

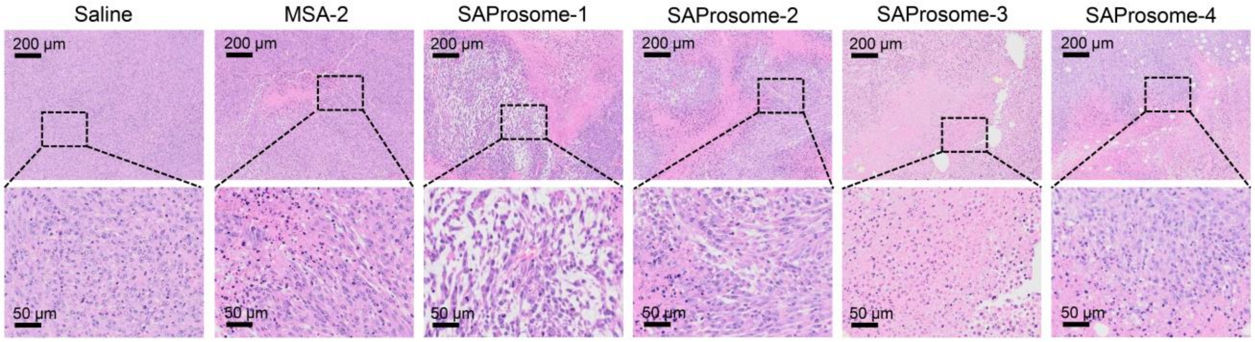

Fig. S15. Representative images of H&E-stained tumor sections from the mice that received different treatments in the MC38 xenograft mouse model. Triplicates were performed independently with similar results.

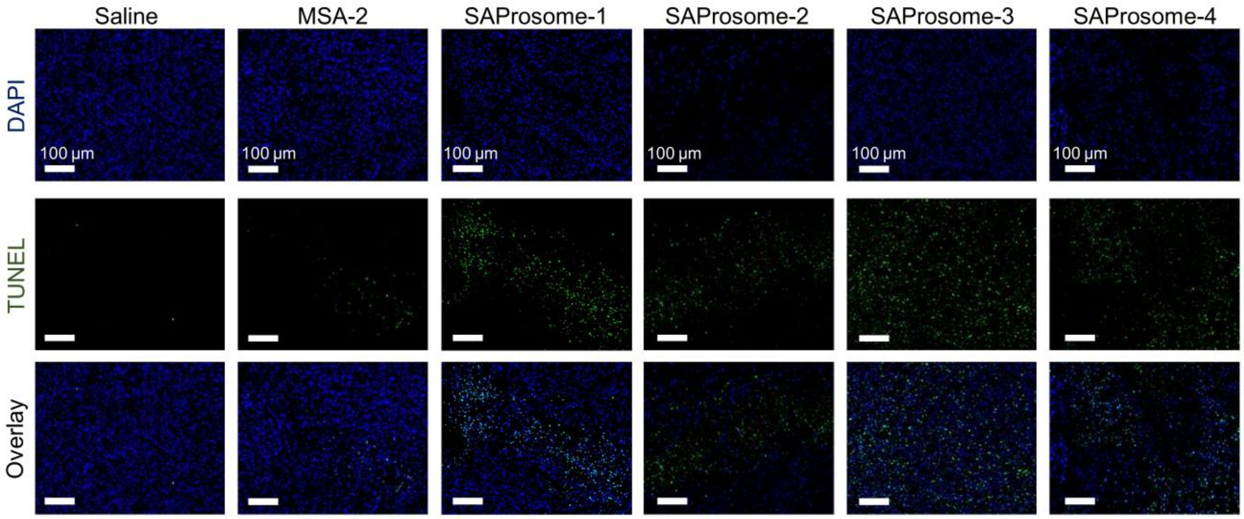

Fig. S16. TUNEL analysis in the excised tumors from the treated groups in the MC38 xenograft mouse model on day 10 post-injection of the drugs. Triplicates were performed independently with similar results.

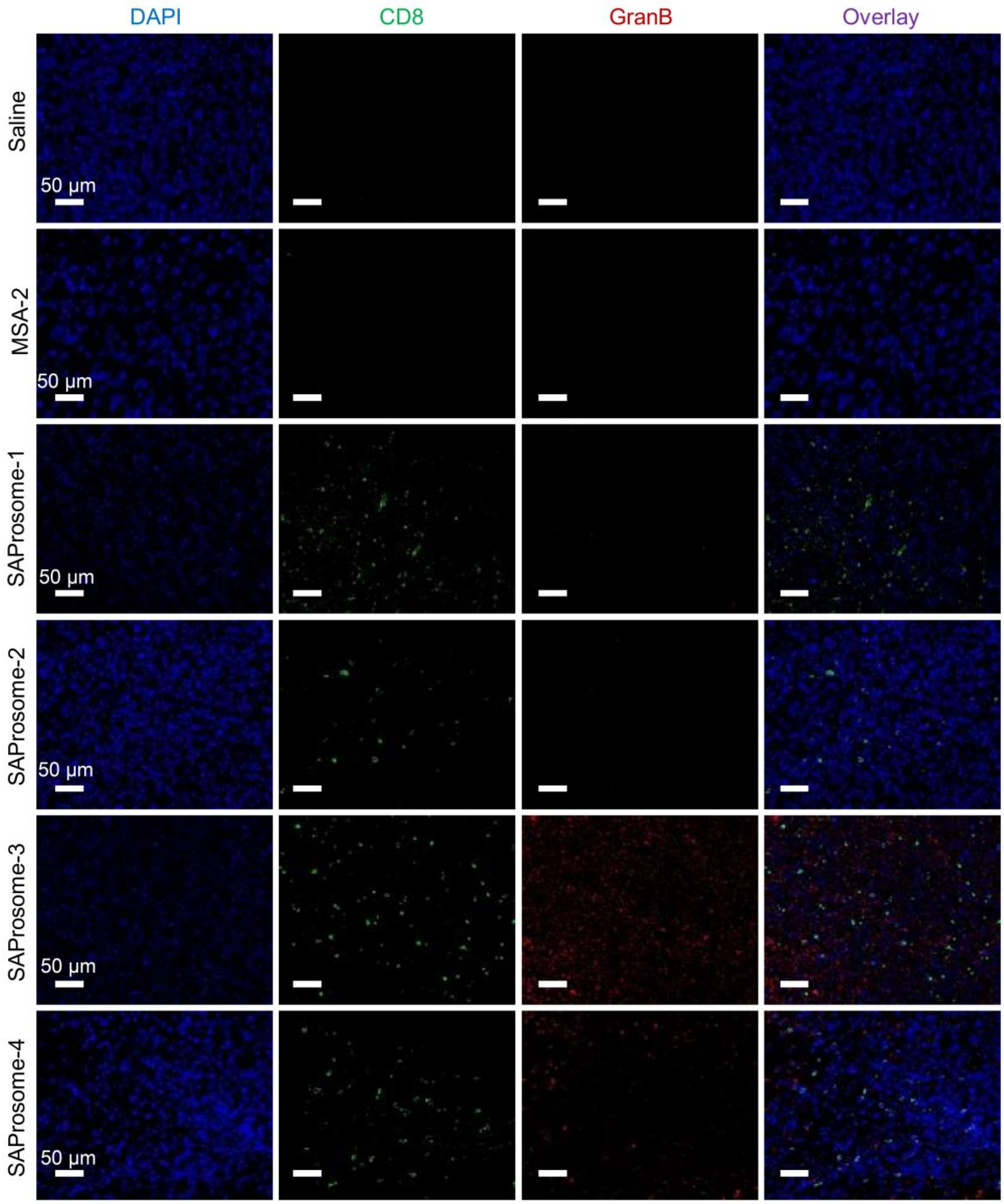

Fig. S17. Immunofluorescence assay of cytotoxic $CD8^+$ T cell in the excised tumors from the treated groups on day 10 post-injection of the drugs in the mouse model of MC38 tumors. Triplicates were performed independently with similar results.

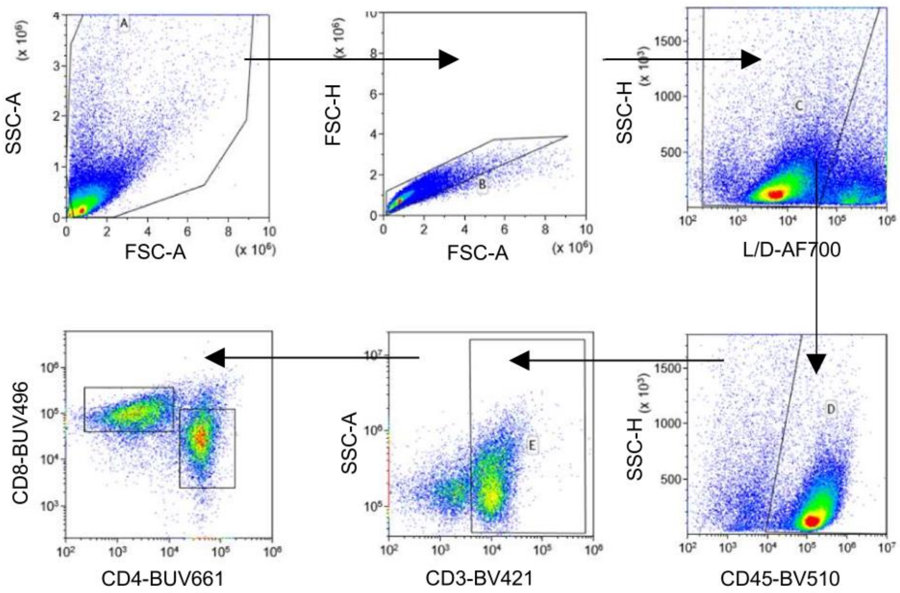

Fig. S18. Gating scheme for flow cytometric analysis of $CD4^+$ and $CD8^+$ T cells.

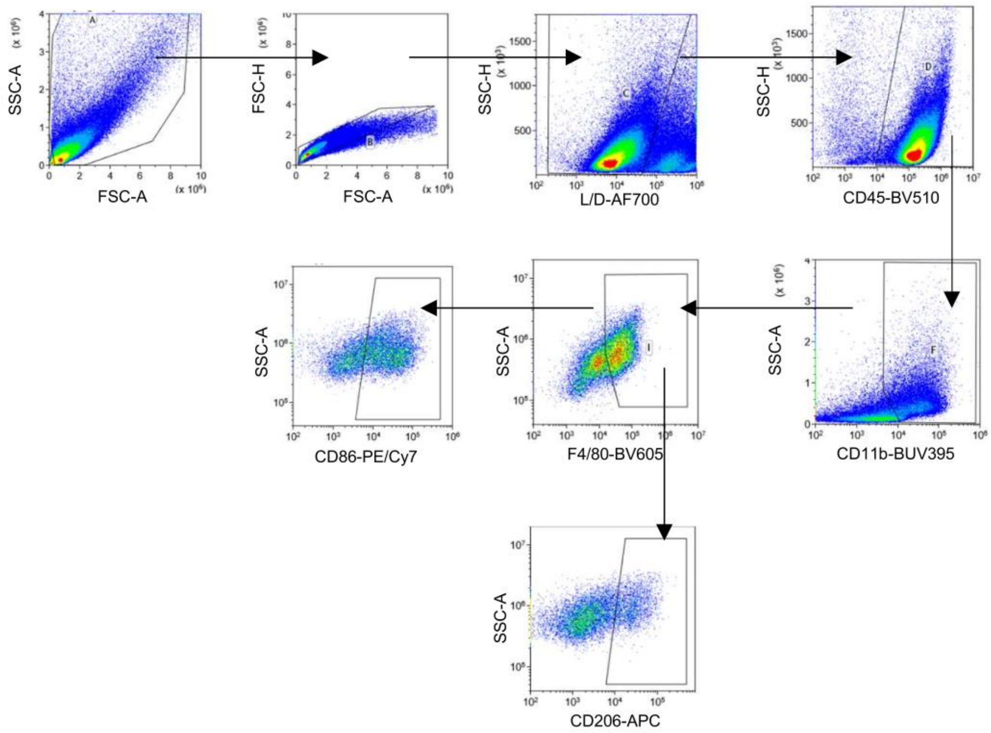

Fig. S19. Gating scheme for analyzing M1-like and M2-like macrophages in the TME using flow cytometry.

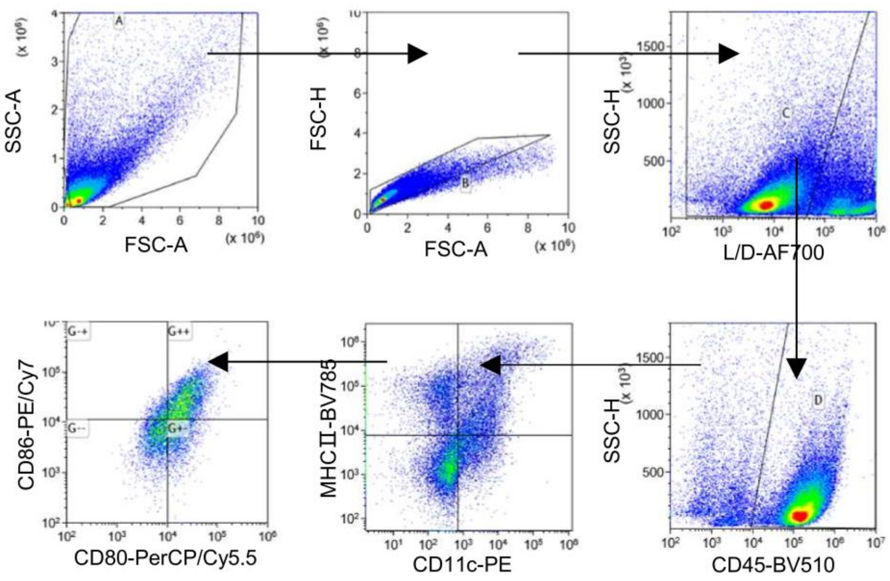

Fig. S20. Flow cytometry gating strategy for analysis of DCs.

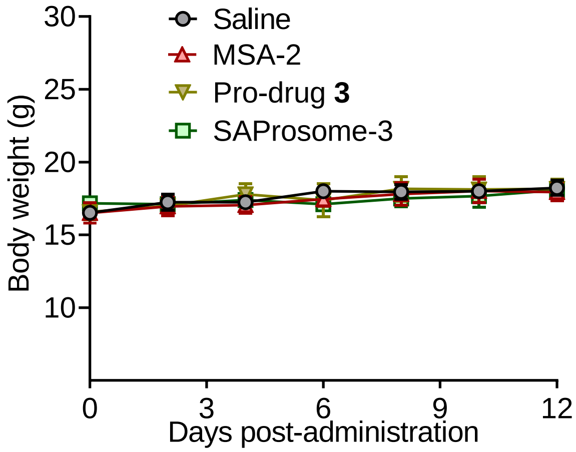

Fig. S21. Measurement of mouse body weight following administration. Two injections were performed on days 0 and 3 in a 4T1 murine breast tumor-bearing mice (n = 5 in each group). Data are presented as mean ± SD.

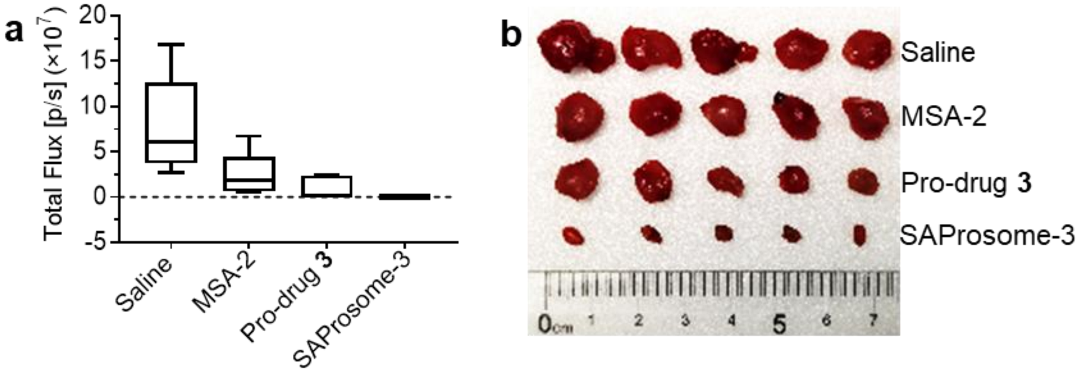

Fig. S22. a Quantitative bioluminescence intensity for whole mouse tumor burden in orthotopic 4T1 tumor-bearing mice from four treatment groups before surgery (n = 5/group). Box plot conveys median (middle line), 25th and 75th percentiles (box), and the minima to maxima range (whiskers). **b** Representative photograph of tumors after treatment. The primary orthotopic tumors were surgically resected on day 12.

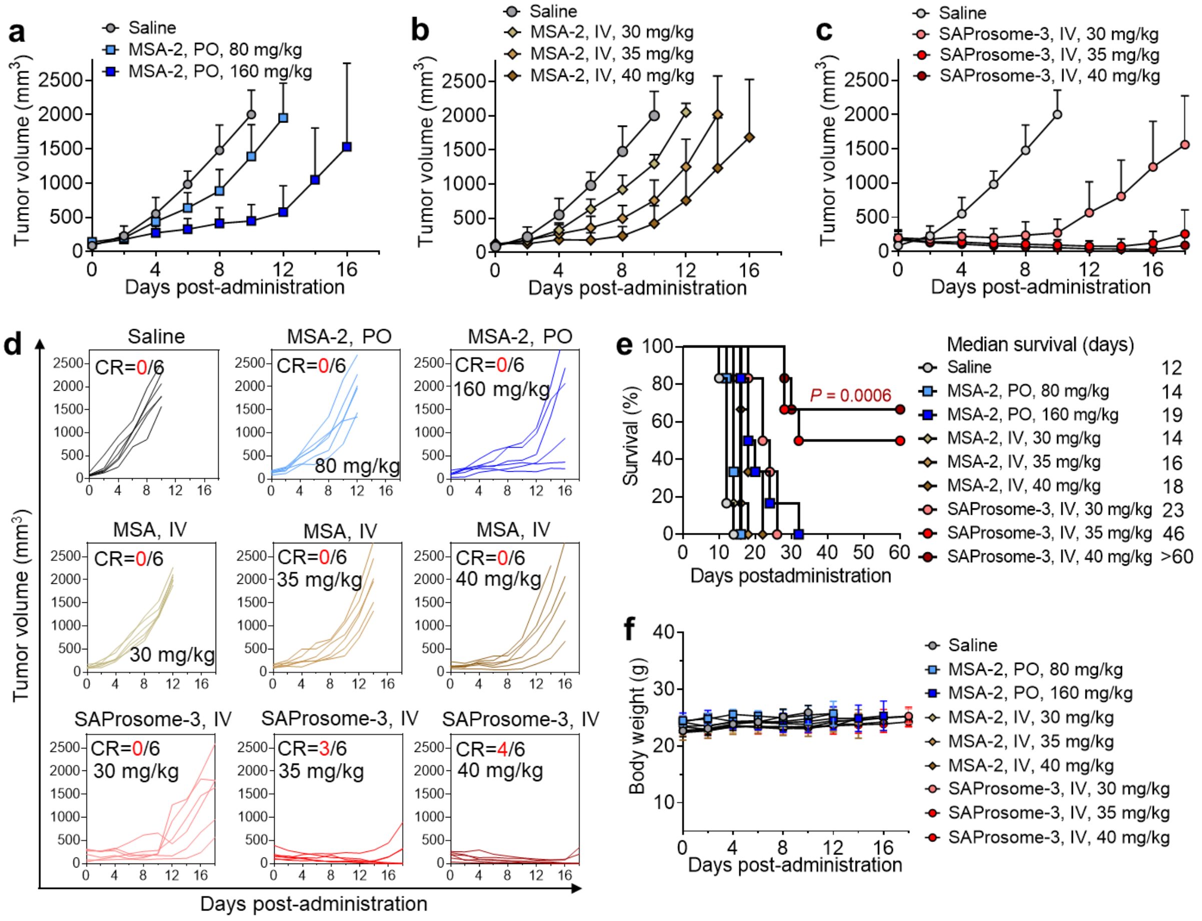

Fig. S23. LLC tumor-bearing mice were treated with free MSA-2 *via* intravenous (IV) administration or oral gavage (PO), and with SAProsome-3 following intravenous injection at MSA-2–equivalent doses as indicated. Tumor growth curves (n = 6) **a-c**, spider plots of individual tumor growth curves **d**, Kaplan-Meier survival curves (log-rank test) **e**, and body weight changes (n = 6) **f**. In panels **a-c** and **f**, data are presented as ± s.d. of mean.

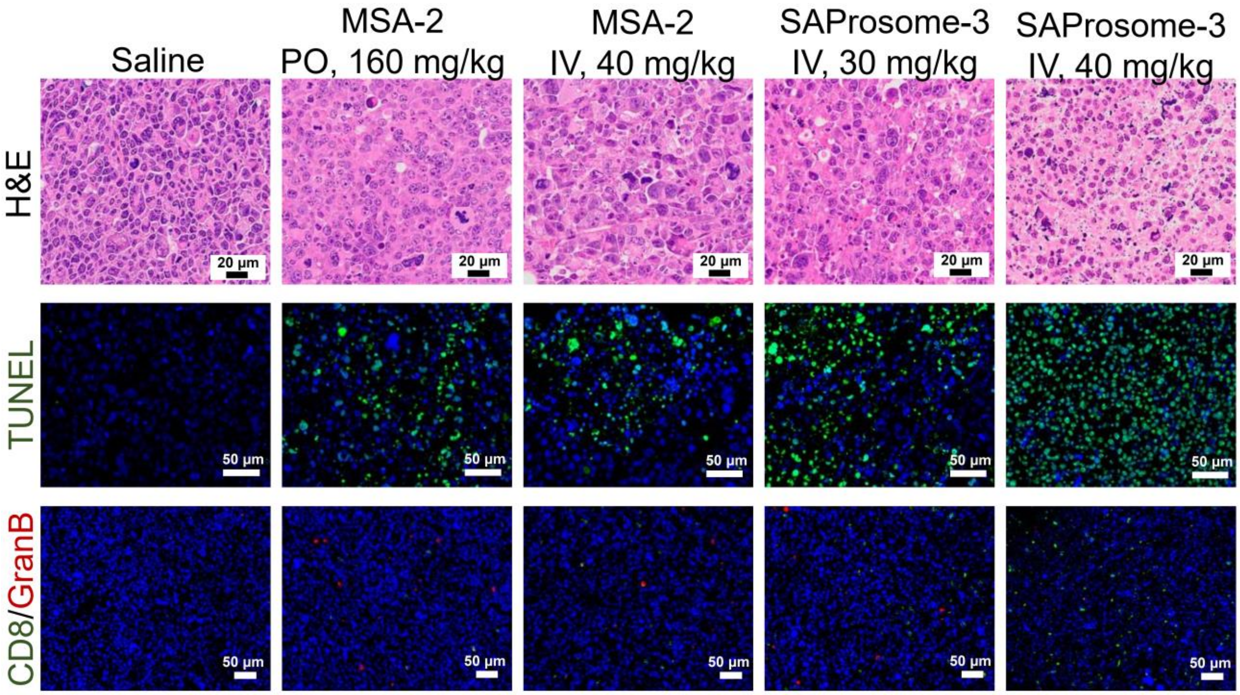

Fig. S24. Representative H&E-staining, TUNEL analysis, and immunofluorescence assay of cytotoxic CD8$^+$ T-cell in excised tumors on day 10 after treatment in the LLC xenograft mouse model. Triplicates were performed independently with similar results.

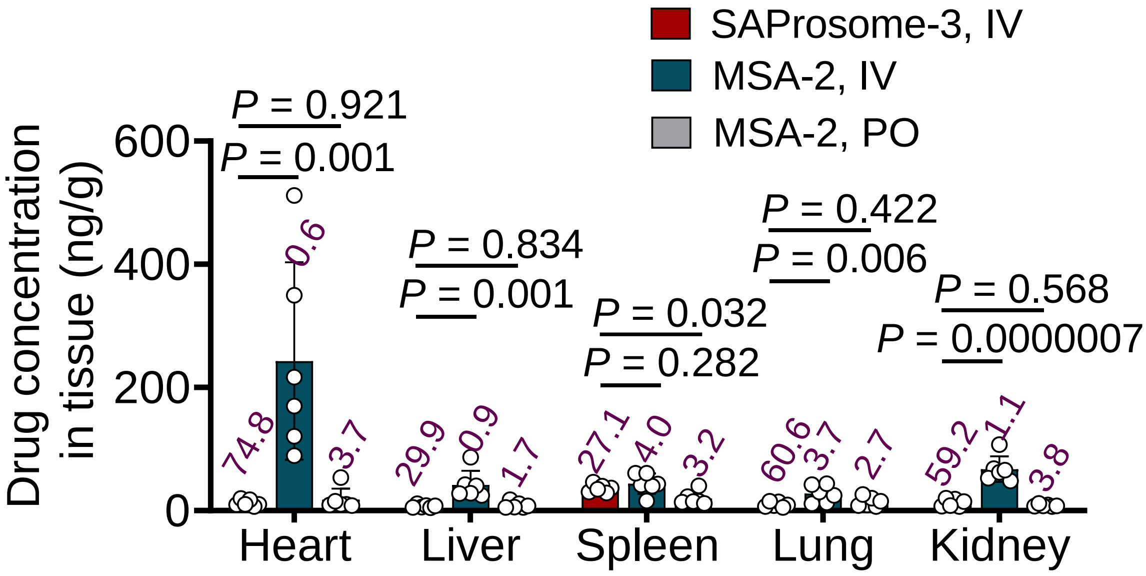

Fig. S25. Mice bearing MC38 xenograft tumors were given a single dose of MSA-2 or SAProsome-3 at a 17.5 mg/kg of MSA-2–equivalent dose. Drug concentration in major organs was determined using HPLC analysis at 8 h after administration. Ratios of drug concentrations in tumors versus other tissues (e.g., heart, liver, spleen, lung and kidney) are shown in purple on the bars. Data represent the means ± SD (n = 6) and statistically analyzed using one-way analysis of variance.

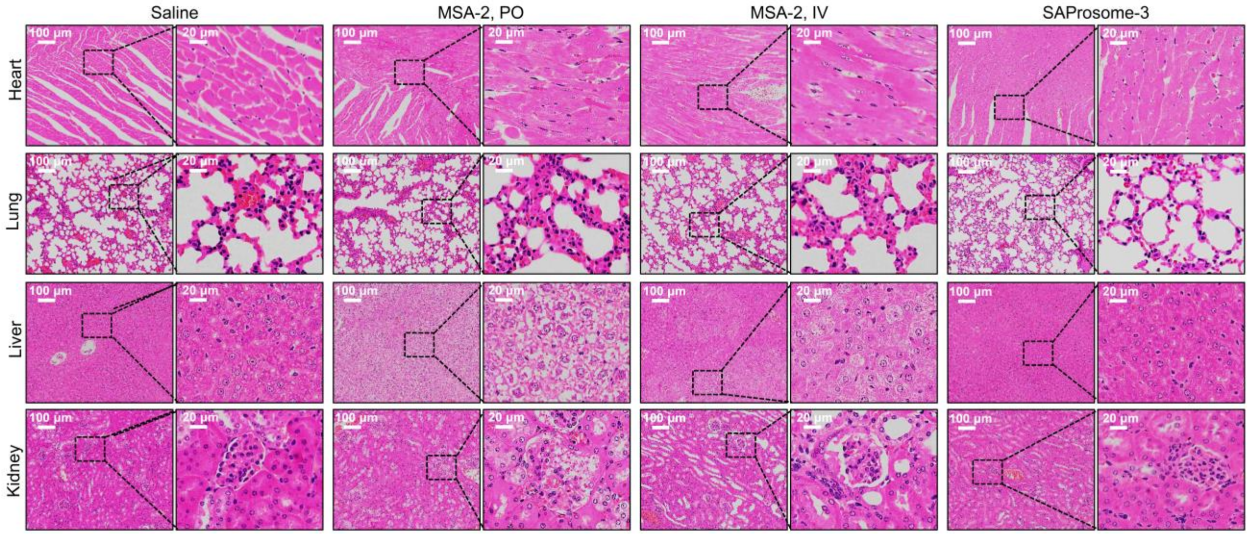

Fig. S26. Representative images of H&E staining of major organs (heart, lung, liver, and kidney) excised from C57BL/6 mice after different treatments. Triplicates were performed independently with similar results.

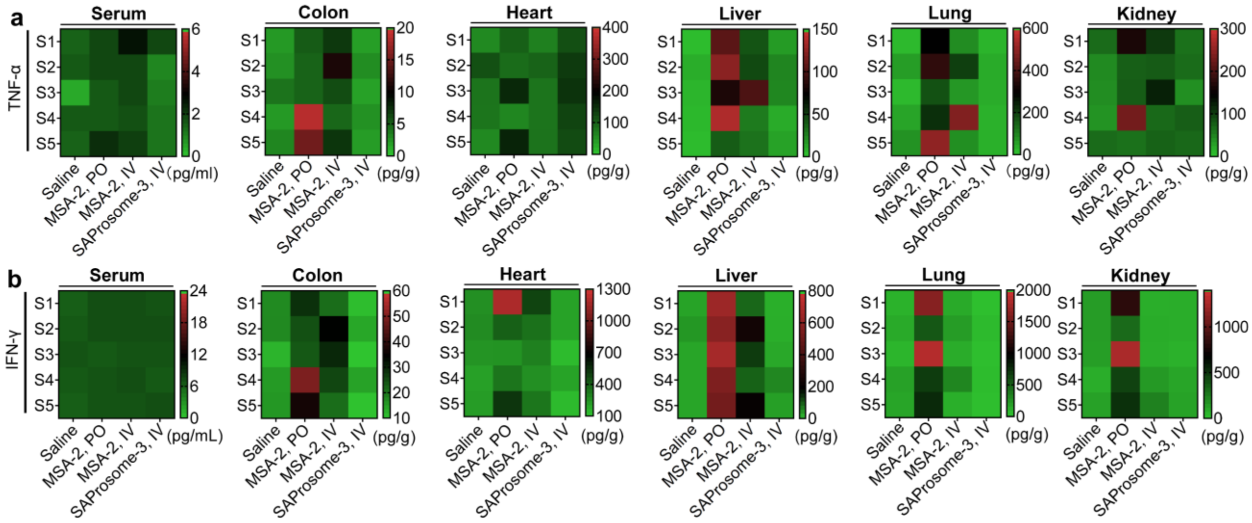

Fig. S27. a-b The levels of TNF-α and IFN-γ in the serum and other tissues were determined using ELISA 6 h after a single administration of free MSA-2 (PO, 240 mg/kg), free MSA-2 (IV, 60 mg/kg) or SAProsome-3 (IV, 45 mg/kg) (n = 5 in each group).

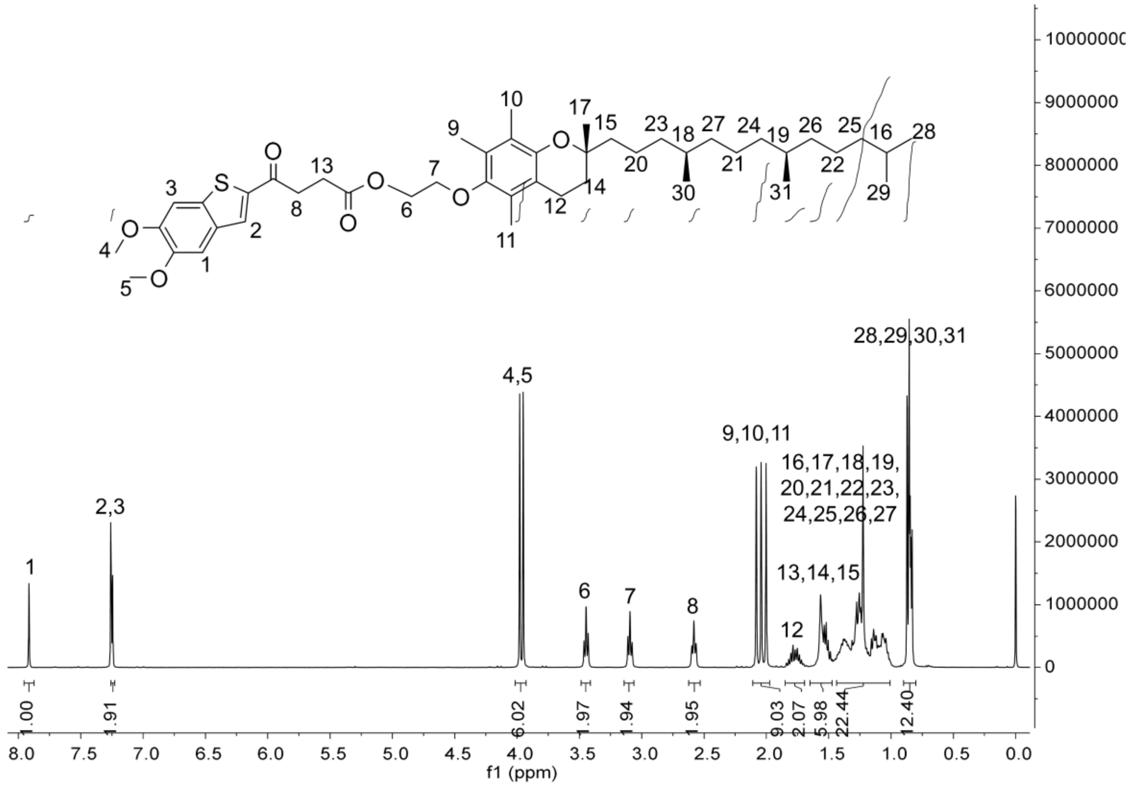

Fig. S28. ^{1}H NMR spectrum of pro-drug **5** in $CDCl_3$.

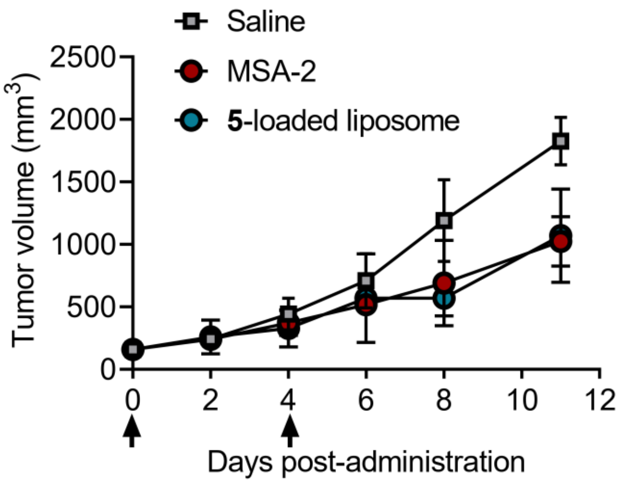

Fig. S29. MC38 tumor-growth kinetics in mice receiving **5**-loaded liposome (IV, MSA-2–equivalent dose of 40 mg/kg) or free MSA-2 (oral administration, 40 mg/kg). Saline intravenously was used as control (n = 3 in each group). Data are presented as ± s.d. of mean.

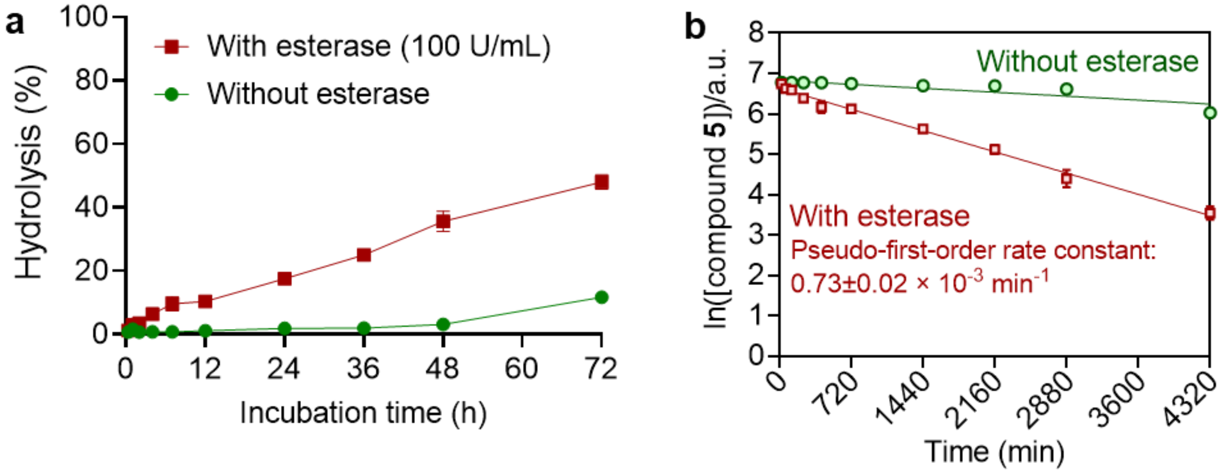

Fig. S30. a *In vitro* hydrolysis profile of pro-drug **5** with or without porcine liver esterase (PLE, 100 unit/mL) in phosphate-buffered saline (PBS) solution at 37°C (n = 3). **b** Interpolation curves and pseudo-first order rate constants for pro-drug **5** hydrolysis (n = 3). Data are presented as ± s.d. of mean.

Table S1. The physicochemical characterizations of STING-activating liposomal vesicles (SAProsomes).

SAProsome	Pro-drug (wt, %)	EPC (wt, %)	Chol (wt, %)	DSPE-PEG$_{2k}$ (wt, %)	EE (%)[a]	MSA-2 DLC (%)[b]	D_H (nm)[c]	Zeta potential (mV)	PDI[d]
SAProsome-1 (pro-drug **1**)	6.79	68.68	9.81	14.72	91.4±2.0	4.48	109.2±2.4	-21.6±0.5	0.27±0.01
SAProsome-2 (pro-drug **2**)	7.01	68.52	9.79	14.68	93.1±1.7	4.55	105.2±1.7	-24.4±1.0	0.26±0.01
SAProsome-3 (pro-drug **3**)	7.23	68.36	9.76	14.65	97.1±0.3	4.74	121.9±2.1	-22.6±0.9	0.19±0.01
SAProsome-4 (pro-drug **4**)	7.66	68.04	9.72	14.58	97.7±0.5	4.75	155.9±3.8	-23.9±0.4	0.14±0.03

a. EE, encapsulation efficiency, as determined by HPLC analysis.

b. DLC, drug loading content.

c. D_H, hydrodynamic diameter.

d. PDI, polydispersity index.

Supplementary Methods

Materials

4-(2-Hydroxypropyl)morpholine and morpholine were purchased from Tokyo Chemical Industry (Shanghai, China). 4-(3-Hydroxypropyl)morpholine was purchased from Aladdin (Shanghai, China). 4-Morpholinobutan-1-ol was obtained from Shanghai Acmec Biochemical Co., Ltd (Shanghai, China). 6-Bromo-1-hexanol was purchased from J&K Chemical (Shanghai, China). 5,6-Dimethoxybenzo[b]thiophene was purchased from Shanghai Renyue Chemical Technology Co., Ltd (Shanghai, China). Porcine liver esterase (PLE) was obtained from Sigma-Aldrich (Shanghai, China). 1,2-Distearoyl-*sn*-glycero-3-phosphoethanolamine-*N*-[methoxy (polyethylene glycol) 2000] (DSPE-PEG_{2k}) and egg-PC was purchased from A.V.T. Pharmaceutical Co., Ltd (Shanghai, China). Recombinant murine Granulocyte/Macrophage Colony-Stimulating Factor (GM-CSF) and mIL4 were obtained from PeproTech Inc. (USA). Anti-IRF3 rabbit monoclonal antibody (mAb, #4302), anti-phospho-IRF3 rabbit mAb (#4947), anti-TBK1 rabbit mAb (#3504), anti-phospho-TBK1 rabbit mAb (#5483), anti-STING rabbit mAb (#13647), anti-phospho-STING rabbit mAb (#50907) and anti-β-actin mouse mAb (#3700) were purchased from Cell Signaling Technology (USA). Anti-mouse IFN-β enzyme-linked immunosorbent assay (ELISA) kit was purchased from Novus Biologicals (USA) and anti-human IFN-β ELISA kit was purchased from R&D Systems (USA). D-Luciferin (potassium Salt) was obtained from Yeasen Biotechnology Co., Ltd (Shanghai, China). The anti-mouse PD-L1 antibody was purchased from BioXcell (USA). All other compounds and solvents were purchased from J&K Chemical (Shanghai, China).

Characterization

All reactions were performed in a dry atmosphere. Thin layer chromatography (TLC) was performed on silica gel 60 F_{254} precoated aluminum sheets (Merck) and visualized using fluorescence quenching. Chromatographic purification was performed using flash column chromatography on silica gel (neutral, Qingdao Haiyang Chemical Co., Ltd). ^{1}H nuclear magnetic resonance (NMR) spectra were recorded on a Bruker 400 spectrometer at 400 MHz in $CDCl_3$ or DMSO-*d6*. Chemical shifts were calibrated to the residual solvent peak or tetramethylsilane (= 0 ppm). High-resolution mass spectrometry (HRMS)-ESI was performed using an AB TripleTOF 5600 plus System (AB SCIEX, Framingham, USA). UV absorption spectra were recorded with a UV-vis spectrometer (Shimadzu, UV-2700). Reverse-phase high-performance liquid chromatography (RP-HPLC) was conducted using a Hitachi Chromaster 5000 system with a YMC-Pack ODS-A column (5 μm, 250 × 4.6 mm). All HPLC runs used linear gradients of acetonitrile (solvent A) and water (solvent B) containing 0.1% trifluoroacetic acid (TFA).

Synthesis of MSA-2

Succinic anhydride (3.1 g, 30.9 mmol) and aluminum trichloride (2.7 g, 20.6 mmol) were dissolved in dichloromethane (DCM, 10 mL) and stirred at 0°C for 1 h. 5,6-dimethoxybenzo[b]thiophene (2.0 g, 10.3 mmol) dissolved in 40 mL of DCM was then added to the above reaction solution dropwise for 30 min. The reaction mixture was then stirred at 43°C overnight. After confirmation of the completed reaction by TLC, the reaction mixture was poured into ice water and the solution pH was adjusted to 10 with sodium hydroxide. The filtrate was obtained by filtration and was further acidified to pH 2 with concentrated hydrochloric acid.

The desired product precipitated as a solid and was collected by filtration. Finally, the precipitate was washed with water and DCM to afford a solid (2.6 g, 85.6 %).

^{1}H NMR (400 MHz, DMSO-*d6*) δ 12.23 (s, 1H), 8.21 (s, 1H), 7.60 (s, 1H), 7.49 (s, 1H), 3.85 (d, *J* = 10.6 Hz, 6H), 3.26 (t, *J* = 6.4 Hz, 2H), 2.61 (t, *J* = 6.4 Hz, 2H).

Synthesis of pro-drug 1

To a solution of 4-(2-hydroxypropyl)morpholine (116 mg, 0.88 mmol) in 3 mL of anhydrous DCM was added MSA-2 (200 mg, 0.68 mmol), *N,N,N',N'*-tetramethyl-O-(benzotriazol-1-yl)uronium tetrafluoroborate (TBTU, 284 mg, 0.88 mmol) and *N,N*-diisopropylethylamine (DIEA, 114 mg, 0.88 mmol). The reaction mixture was stirred at 43°C overnight and the solvent was removed by evaporation. The residue was dissolved in DCM and washed with 5% citric acid, saturated $NaHCO_3$ and brine. The organic layer was dried over anhydrous Na_2SO_4, filtered, and evaporated under vacuum. The crude product was further purified by flash column chromatography on silica gel to afford the pro-drug **1** as a pink solid (188 mg, 68%).

^{1}H NMR (400 MHz, $CDCl_3$) δ 7.89 (s, 1H), 7.25 (d, *J* = 3.4 Hz, 2H), 4.26 (t, *J* = 5.8 Hz, 2H), 3.97 (d, *J* = 9.8 Hz, 6H), 3.70 (t, *J* = 4.6 Hz, 4H), 3.33 (t, *J* = 6.8 Hz, 2H), 2.81 (t, *J* = 6.8 Hz, 2H), 2.65 (t, *J* = 5.8 Hz, 2H).

Synthesis of pro-drug 2

To a solution of 4-(3-hydroxypropyl)morpholine (128 mg, 0.88 mmol) in 3 mL of anhydrous DCM was added MSA-2 (200 mg, 0.68 mmol), TBTU (284 mg, 0.88 mmol) and DIEA (114 mg, 0.88 mmol). The reaction mixture was stirred at 43°C overnight and the solvent was removed by evaporation. The residue was dissolved in DCM and washed with 5% citric

acid, saturated $NaHCO_3$ and brine. The organic layer was dried over anhydrous Na_2SO_4, filtered, and evaporated under vacuum. The crude product was further purified by flash column chromatography on silica gel to give the pro-drug **2** as a yellow solid (178 mg, 62%).

^{1}H NMR (400 MHz, $CDCl_3$) δ 7.90 (s, 1H), 4.17 (t, *J* = 6.4 Hz, 2H), 3.97 (d, *J* = 9.8 Hz, 6H), 3.71 (t, *J* = 4.8 Hz, 4H), 3.32 (t, *J* = 6.8 Hz, 2H), 2.79 (t, *J* = 6.8 Hz, 2H), 2.44 (s, 6H), 1.91–1.79 (m, 2H).

Synthesis of pro-drug 3

To a solution of 4-morpholinebutanol (140 mg, 0.88 mmol) in 3 mL of anhydrous DCM was added MSA-2 (200 mg, 0.68 mmol), TBTU (284 mg, 0.88 mmol) and DIEA (114 mg, 0.88 mmol). The reaction mixture was stirred at 43°C overnight and the solvent was removed by evaporation. The residue was dissolved in DCM and washed with 5% citric acid, saturated $NaHCO_3$ and brine. The organic layer was dried over anhydrous Na_2SO_4, filtered, and evaporated under vacuum. The crude product was further purified by flash column chromatography on silica gel to give the pro-drug **3** as a pink solid (258 mg, 85%).

^{1}H NMR (400 MHz, $CDCl_3$) δ 7.89 (s, 1H), 7.25 (d, *J* = 2.8 Hz, 2H), 4.13 (t, *J* = 6.4 Hz, 2H), 3.97 (d, *J* = 9.8 Hz, 6H), 3.72 (t, *J* = 4.6 Hz, 4H), 3.32 (t, *J* = 6.8 Hz, 2H), 2.78 (t, *J* = 6.8 Hz, 2H), 2.53–2.30 (m, 6H), 1.67 (dd, *J* = 8.0, 6.1 Hz, 2H).

Synthesis of pro-drug 4

6-Morpholino-1-hexanol was synthesized *via* a simple substitution reaction. Briefly, morpholine (0.6 g, 6.6 mmol), 6-Bromo-1-hexanol (1.0 g, 5.5 mmol) and K_2CO_3 (0.7 g, 3.3 mmol) were mixed in 25 mL anhydrous acetonitrile, and stirred at 80°C overnight. The solvent was removed by evaporation. The mixture was cooled to room temperature and then poured into

water (100 mL). The solution was extracted with DCM. The organic layers were collected and evaporated under vacuum to obtain the residue that was further purified by flash column chromatography on silica gel to give the product as a colorless oil (879 mg, 85%).

^{1}H NMR (400 MHz, $CDCl_3$) δ 3.77–3.68 (m, 4H), 3.63 (t, *J* = 6.6 Hz, 2H), 2.53–2.38 (m, 4H), 2.37–2.29 (m, 2H), 1.62–1.45 (m, 4H), 1.37 (qtd, *J* = 13.0, 6.8, 2.8 Hz, 4H).

To a solution of 6-morpholino-1-hexanol (162 mg, 0.88 mmol) in 3 mL of anhydrous DCM was added MSA-2 (200 mg, 0.68 mmol), TBTU (284 mg, 0.88 mmol) and DIEA (114 mg, 0.88 mmol). The reaction mixture was stirred at 43°C overnight and the solvent was removed by evaporation. The residue was dissolved in DCM and washed with 5% citric acid, saturated $NaHCO_3$ and brine. The organic layer was dried over anhydrous Na_2SO_4, filtered, and evaporated under vacuum. The crude product was further purified by flash column chromatography on silica gel to give the pro-drug **4** as a pink solid (227 mg, 72%).

^{1}H NMR (400 MHz, $CDCl_3$) δ 7.89 (s, 1H), 7.25 (d, *J* = 2.6 Hz, 2H), 4.10 (t, *J* = 6.8 Hz, 2H), 3.97 (d, *J* = 9.8 Hz, 6H), 3.73 (t, *J* = 4.6 Hz, 4H), 3.31 (t, *J* = 6.8 Hz, 2H), 2.78 (t, *J* = 6.8 Hz, 2H), 2.44 (s, 4H), 2.33 (t, *J* = 7.8 Hz, 2H), 1.66–1.60 (m, 2H), 1.47 (s, 2H), 1.40–1.30 (m, 4H).

Synthesis of pro-drug 5

MSA-2 (120 mg, 0.41 mmol) was added to anhydrous DCM containing α-tocopherol (193 mg, 0.45 mmol), 4-dimethylaminopyridine (55 mg, 0.45 mmol) and 1-(3-dimethylaminopropyl)-3-ethylcarbodiimide (70 mg, 0.45 mmol). The reaction mixture was stirred at 43°C overnight and the solvent was removed by evaporation. The residue was dissolved in DCM and washed with 5% citric acid, saturated $NaHCO_3$ and brine. The organic layer was dried over anhydrous

Na_2SO_4, filtered, and evaporated under vacuum. The crude product was further purified by flash column chromatography on silica gel to give the pro-drug **5** as a yellow solid (262 mg, 89.9%).

^1H NMR (400 MHz, $CDCl_3$) δ 7.25 (d, *J* = 4.2 Hz, 2H), 3.96 (d, *J* = 11.4 Hz, 6H), 3.45 (t, *J* = 6.8 Hz, 2H), 3.09 (t, *J* = 6.8 Hz, 2H), 2.58 (t, *J* = 6.8 Hz, 2H), 2.11–1.97 (m, 9H), 1.76 (dp, *J* = 20.2, 6.6 Hz, 2H), 1.44–1.01 (m, 22H), 0.85 (m, 12H).

Determination of the drug encapsulation efficiency and drug loading content

The encapsulation efficiency (EE) and drug loading content (DLC) of SAProsomes were evaluated using HPLC. Briefly, after preparing the SAProsomes, the solutions were centrifuged at 100000 g for 30 minutes. The supernatant was diluted with a solution of acetonitrile/water (CH_3CN/H_2O, v/v, 1/1), and the drug contents were determined using HPLC. UV detection of MSA-2 was performed at wavelengths of 326 nm. The EE and DLC values were calculated using equations (1) and (2):

$$EE\ (\%) = W_{\text{pro-drug in SAProsomes}}/W_{\text{initial pro-drug added}} \times 100\% \qquad (1)$$

$$DLC\ (\%) = W_{\text{drug}}/W_{\text{total}} \times 100\% \qquad (2)$$

where $W_{\text{pro-drug in SAProsomes}}$, $W_{\text{initial pro-drug added}}$, W_{drug} and W_{total} represent the weights of total pro-drugs formulated into SAProsomes, the initial pro-drug added for encapsulation, total drugs formulated into SAProsomes and total SAProsomes, respectively.

Characterization of SAProsomes

To measure the particle size distribution and zeta potential, particles were diluted in phosphate-buffered saline (PBS) and characterized using a Malvern Nano ZS. For transmission electron microscopy (TEM), particles were drop cast onto a carbon film-coated 200-mesh copper

grid, stained with a 2% solution of uranyl acetate for 30 s and imaged on a 120 kV Transmission Electron Microscope (Tecnai G2 Spirit, Thermo FEI).

For Cryogenic TEM, some 3 μl of sample (at 2 mg/mL of MSA-2 concentration) was dropped on a lacey copper grid coated with a continuous carbon film. The blotter was used to remove excess sample without damaging the carbon layer before plunge freezing. The frozen grid was mounted on a cryo-transfer holder, and a transfer workstation with liquid nitrogen was used to maintain the specimen and holder under frozen conditions before imaging. The sample was imaged on Talos F200C (200 kV, Thermo FEI).

Hydrolysis rate of MSA-2 pro-drugs

The *in vitro* hydrolysis of the MSA-2 pro-drugs in the presence or absence of porcine liver esterase (PLE) was evaluated by HPLC analysis. Briefly, 10 mL aqueous solutions of pro-drugs **1-4** (50 μg/mL) were incubated with or without PLE (50 units/mL) at 37°C. At various time intervals, aliquot samples (100 μL) were collected and subjected to analytical RP-HPLC at a flow rate of 1.0 mL/min using acetonitrile/water as the mobile phase. UV detection was performed at a wavelength of 326 nm. The amounts of compounds were calculated by establishing standard curves. The hydrolysis rate of the pro-drugs was calculated as a function of the incubation time.

Stability of SAProsomes loading MSA-2 pro-drugs

The SAProsome 1-4 (0.2 mg/mL, MSA-2 equivalent) in PBS (pH 7.4) or supplemented with 20% (v/v) fetal bovine serum (FBS) were incubated at 37°C. Dynamics light scattering analysis was utilized to determine the particle size and polydisperse index (PDI) during incubation.

<u>Modulation of IFN-I response by SAProsomes *in vitro*</u>

Murine bone marrow-derived dendritic cells (BMDCs) were isolated and cultured. To assess the effect of SAProsomes for modulating the IFN-I response, we seeded 1×10^5 BMDCs or THP1 cells per well in 96-well plates. Free MSA-2 or different SAProsomes were added at the indicated MSA-2 concentrations for 6 h incubation. Total RNAs were extracted from cells and then RNA quantity and quality were confirmed using the NanoDrop (NanoDrop one, thermo scientific) system. Genomic DNA was removed and cDNA was synthesized using an iScript gDNA clear cDNA synthesis kit (Bio-Rad). Bio-Rad SsoAdvanced universal SYBR green supermix and real-time system (CFX96 Touch, Bio-Rad) were used for PCR analysis. β-actin was used as an endogenous normalization control to obtained relative expression data. The DNA primers used were as follows:

mouse IFNβ1: ATGAGTGGTGGTTGCAGGC, TGACCTTTCAAATGCAGTAGATTCA;

mouse TNFα: CCTGTAGCCCACGTCGTAG, GGGAGTAGACAAGGTACAACCC;

mouse CXCL10: GGAGTGAAGCCACGCACAC, ATGGAGAGAGGCTCTCTGCTGT;

mouse β-actin: ACACCCGCCACCAGTTCGC, ATGGGGTACTTCAGGGTCAGGATA;

human IFNβ1: GCTTCTCCACTACAGCTCTTTC, CAGTATTCAAGCCTCCCATTCA;

human TNFα: CCAGGGACCTCTCTCTAATCA, TCAGCTTGAGGGTTTGCTAC;

human CXCL10: CCATTCTGATTTGCTGCCTTATC, TACTAATGCTGATGCAGGTACAG;

human β-actin: ATCAAGATCATTGCTCCTCCTGAG, CTGCTTGCTGATCCACATCTG.

To examine the expression of STING, IRF3, and TBK1, and corresponding phosphorylation, human THP1 cells were seeded at a density of 2×10^6 cells per well in 6-well

plates, and treated with free MSA-2 or SAProsome-3 at a concentration of 40 μM. After 6 hours of incubation, cells were collected and lysed in RIPA lysis buffer. The protein concentration was determined using a BCA assay (Beyotime Biotechnology, Shanghai). Samples were run on an SDS-PAGE gel and transferred onto a nitrocellulose membrane. The membranes were washed and incubated with primary antibodies (including anti-IRF3 rabbit mAb, anti-phospho-IRF3 rabbit mAb, anti-TBK1 rabbit mAb, anti-phospho-TBK1 rabbit mAb, anti-STING rabbit mAb, anti-phospho-STING rabbit mAb and anti-β-actin mouse mAb, Cell Signaling Technology, 1000 ×) overnight at 4°C, followed by blotting with HRP-conjugated secondary antibodies. The protein bands were visualized using the ChemiDoc MP system (Bio-Rad, USA) and an immobile western Chemiluminescent HRP Substrate Kit. Protein loading was normalized for equal amounts of actin.

To further determine the IFN-β expression, mouse BMDCs and human THP1 cells were seeded in 12-well plates (8×10^5 cells per well), and free MSA-2 or different SAProsome at series of concentrations were added. After incubation at predetermined time points, the supernatants were collected and secreted IFNβ was quantified with IFNβ ELISA kit.

Activation of BMDC and cross-presentation of antigens by BMDC

BMDCs were isolated as mentioned above. To measure the maturation level of DCs after various treatments, BMDCs were seeded into 12-well plates at a density of 5×10^5 cells/well, followed by SAProsomes or free MSA-2 treatment at an MSA-2 dose of 40 μM. After incubation for 6 h, cells were collected and measured for expression of activation markers using APC anti-mouse CD45 (Biolegend, 103112, 80 ×), PE/Cy7 anti-mouse CD11c (Biolegend, 117318, 40 ×), FITC anti-mouse CD80 (Biolegend, 104706, 80 ×), Percy/Cy5.5 anti-mouse CD86 (Biolegend,

105028, 80 ×) and Brilliant Violet 421 anti-mouse I-A/I-E (Biolegend, 107632, 80 ×) by flow cytometry using LX CytExpert 2.4 software.

To detect cross-presentation of antigens by DC, BMDCs were treated with free MSA-2 or SAProsomes for 6 hours at an MSA-2 dose of 40 μM and incubated overnight with 40 nM of $OVA_{257-264}$ (SIINFEKL). After incubation, the cells were also stained with an APC-labeled 25-D1.16 monoclonal antibody directed against SIINFEKL-H-2Kb complexes (Biolegend, 141606, 20 ×). The mean fluorescence intensity (MFI) of APC within DCs engulfing tumor antigens by flow cytometry was assessed using LX CytExpert 2.4 software.

<u>Cytolytic analysis of splenocytes primed by BMDCs</u>

To assess the cytolytic capacity of $CD8^+$ T cells induced by stimulated DCs, B16F10 or B16F10-OVA cells were used as target cells. B16F10 or B16F10-OVA cells were seeded to 48-well plates at a density of 1×10^4 cells/well, which were pretreated with 5µg/mL of mitomycin C to prevent cell proliferation. BMDCs were incubated with indicated formulations for 6 hours at an MSA-2 dose of 40 μM and then treated overnight with 40 nM of $OVA_{257-264}$ (SIINFEKL). Meanwhile, spleens from OT1 mice were harvested and processed to obtain splenocytes with single cell suspensions. Then, the splenocytes and BMDCs were mixed at the cell number ratio of 10:5 (BMDCs = 5×10^4) and co-cultured with B16F10 or B16F10-OVA cells. BMDCs were co-cultured with B16F10 or B16F10-OVA as the negative control. After incubation for 48 h, the supernatant was collected and centrifuged to remove cell debris for the measurement of lactate dehydrogenase (LDH) release by LDH cytotoxicity assay kit (#BC0685, Solarbio). The cell death can be represented by the percentage of LDH release that was calculated as follows: (sample LDH – spontaneous LDH)/ (total LDH - spontaneous LDH) ×100%.

<u>Evaluation of immunoregulatory activity in tumor-bearing mice</u>

Mice were subcutaneously inoculated with MC38 cells (5×10^6 cells) into the right flank. On reaching sizes of ~200 mm^3, the animals were randomized into three groups (n = 8 in each group). Mice were treated with three injections of SAProsome3 intravenously at an MSA-2 dose of 35 mg/kg on days 0, 4, and 8, while free MSA-2 were taken by oral administration for comparison. Mice were euthanized, and tumors, tumor-draining lymph nodes (TDLNs) were harvested.

For flow cytometric analysis, the tumors and TDLNs were harvested, mechanically dissociated with a scissor, and digested in a solution of 125 μg/mL Deoxyribonuclease I (Sigma-Aldrich) and 1 mg/mL Collagenase IV (Sigma-Aldrich) in RPMI 1640 media for 30 min at 37 °C. After digestion, tumors and lymph nodes were strained through a 70 μm cell strainer and were diluted to a concentration of 2×10^7 cells per mL in PBS containing 2% fetal bovine serum (FBS) for staining with fluorescent antibodies. 100 μl of cell suspension for each flow test was transferred into the tube and treated with TruStain FcX (#101320, Biolegend) according to the manufacturer's specifications. Samples were stained with several panels of the antibodies including BV510 anti-mouse CD45 (Biolegend, 103138, 80 ×), BV421 anti-mouse CD3 (Biolegend, 100228, 80 ×), BUV661 anti-mouse CD4 (BD Biosciences, 612974, 40 ×), BUV496 anti-mouse CD8a (BD Biosciences, 750024, 40 ×), BUV395 anti-mouse CD11b (BD Biosciences, 563553, 40 ×), PE anti-mouse CD11c (BD Biosciences, 557401, 40 ×), FITC anti-mouse Ly6C (BD Biosciences, 553104, 40 ×), APC/Cy7 anti-mouse Ly6G (BD Biosciences, 560600, 40 ×), BV605 anti-mouse F4/80 (BD Biosciences, 743281, 40 ×), PE/Cy7 anti-mouse CD86 (Biolegend, 105014, 80 ×), Percy/Cy5.5 anti-mouse CD80 (Biolegend, 104722, 80 ×), PE/CF594 anti-mouse NK1.1 (BD Biosciences, 562864, 40 ×), BV786 anti-mouse I-A/I-E (BD Biosciences, 743875, 80 ×). BV, brilliant violet; PE, phycoerythrin; Cy, cyanine; APC,

allophycocyanin. Cells were washed twice, suspended in PBS containing 2% FBS and then subjected into a flow cytometer using LX CytExpert 2.4 software. For intracellular cytokine analysis, 10^6 cells were seeded in a 6-well plate in Dulbecco's modified Eagle's medium containing 10% FBS and supplemented with a PMA/ionomycin/Brefeldin A cocktail (Biolegend) according to the manufacturer's specification. After 4 h, the cells were washed, stained with antibodies against CD45, CD3, CD4, CD8α and CD107a (Biolegend), fixed with fixation buffer (Biolegend) and subsequently stained intracellularly with antibodies against CD206, TNF-α and IFN-γ in Intracellular Staining Permeabilization Wash Buffer (Biolegend).

Establishment of LLC murine model for antitumor efficacy study

To conduct antitumor efficacy studies, a murine model of LLC was established. The experiment involved subcutaneously inoculating mice with 5×10^5 LLC into the right flank. Tumor size was measured every 2 days using a digital caliper, and tumor volume was calculated as $0.5 \times \text{length} \times \text{width}^2$. Once the tumor size reached ~100 mm^3, the animals were randomized into eleven groups with n = 6 mice in each group. Three injections of different SAProsomes were administered *via* the tail vein at the dose of 30, 35, or 40 mg/kg on days 0, 4, and 8. Additionally, free MSA-2 was administered orally at 80 or 160 mg/kg dose and intravenously at 30, 35, or 40 mg/kg dose. Tumor growth and body weight were monitored and recorded every 2 days. The mice were euthanized via CO_2 inhalation at the end of the study.

Evaluation of *in vivo* drug toxicity

We evaluated the acute inflammatory toxicity of free MSA-2 and SAProsome-3 by measuring multiple cytokines/chemokines in the serum and other tissues from healthy C57BL/6 mice (4-5 weeks old). To do this, mice were randomized into four groups (n = 5 in each group). Single intravenous injection of SAProsome-3 at an MSA-2–equivalent dose of 45 mg/kg were

administered, while free MSA-2 was taken by oral administration at a dose of 240 mg/kg or by intravenous injection at a dose of 60 mg/kg for comparison. At 6 hours after administration, blood, colon, heart, liver, lung, and kidney were harvested, and the tissues were homogenized using a TissueLyser II (Qiagen) in RIPA lysis buffer. The levels of IL-6, TNF-α, and IFN-γ in the serum and tissue homogenate were determined by an ELISA kit.

To further evaluate the potential hematological side effects, healthy C57BL/6 mice were intravenously administered with saline or SAProsome3 at an MSA-2–equivalent dose of 45 mg/kg every three days for three consecutive times. Free MSA-2 was also administered through intravenous injection at a dose of 60 mg/kg or by oral gavage at a dose of 240 mg/kg for comparison. Blood samples (n = 4 in each group) were collected on day 8 for blood biochemistry analysis to assess the influence on liver and kidney function. Hepatorenal parameters, including aspartate aminotransferase (AST), alanine aminotransferase (ALT), uric acid (UA), and blood urea nitrogen (BUN), were measured. Additionally, major organs including liver, heart, kidney, and lung were excised on day 8 and subjected to H&E staining to observe any histological changes.

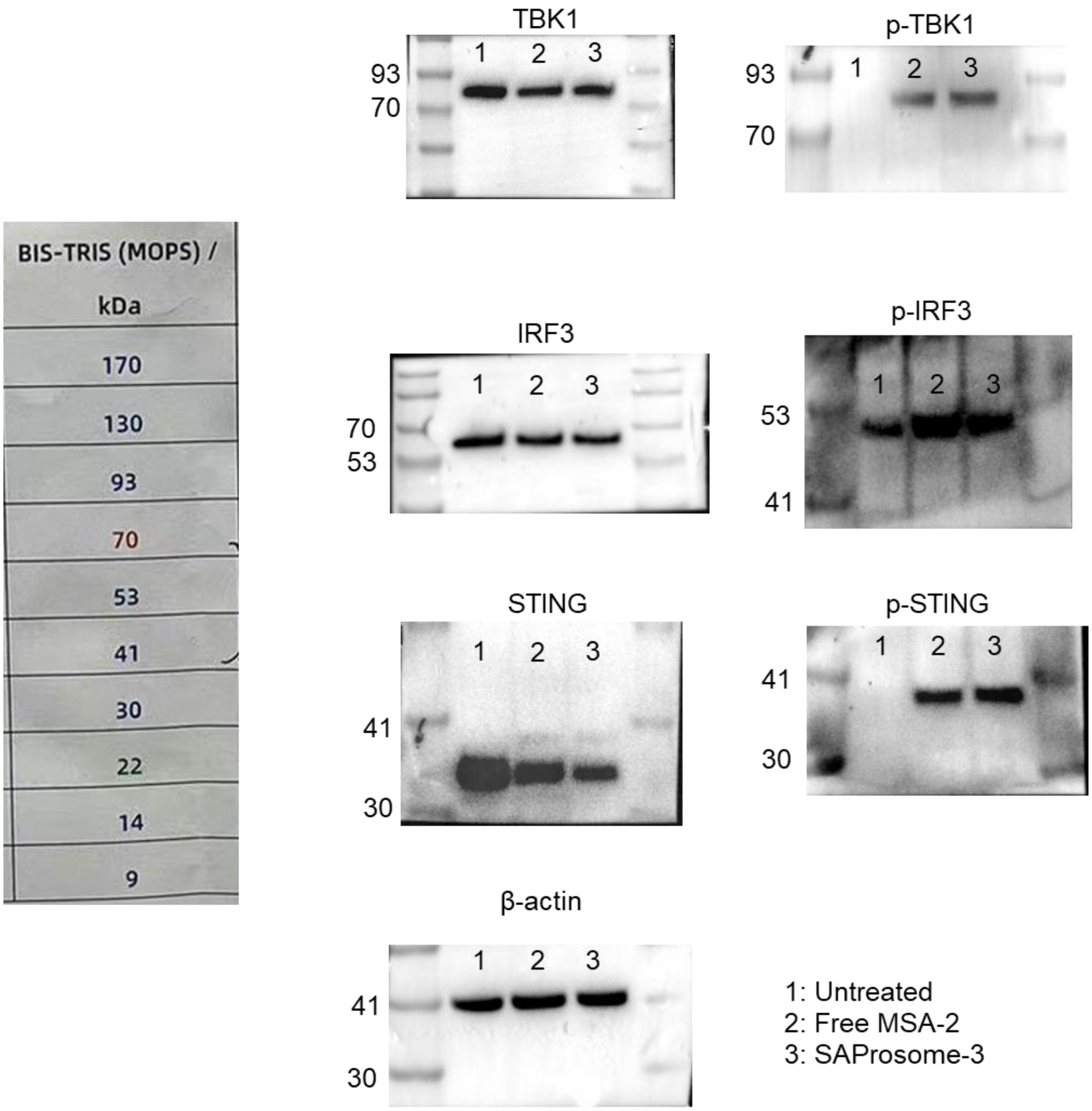

Uncropped scans of blots in Supplementary Figure 13a were provided here.
